# Saccadic Compression of Symbolic Numerical Magnitude

**DOI:** 10.1371/journal.pone.0049587

**Published:** 2012-11-15

**Authors:** Paola Binda, M. Concetta Morrone, Frank Bremmer

**Affiliations:** 1 Department of Translational Research on New Technologies in Medicine and Surgery, Università di Pisa, Pisa, Italy; 2 Department of Psychology, University of Washington, Seattle, Washington, United States of America; 3 Scientific Institute Stella Maris, Pisa, Italy; 4 Department of Neurophysics, Philipps-Universität Marburg, Marburg, Germany; Bielefeld University, Germany

## Abstract

Stimuli flashed briefly around the time of saccadic eye movements are subject to complex distortions: compression of space and time; underestimate of numerosity. Here we show that saccadic distortions extend to abstract quantities, affecting the representation of symbolic numerical magnitude. Subjects consistently underestimated the results of rapidly computed mental additions and subtractions, when the operands were briefly displayed before a saccade. However, the recognition of the number symbols was unimpaired. These results are consistent with the hypothesis of a common, abstract metric encoding magnitude along multiple dimensions. They suggest that a surprising link exists between the preparation of action and the representation of abstract quantities.

## Introduction

Saccadic eye movements cause visual images to rapidly shift across the retina. While early work suggested that stable perception is achieved by ‘subtracting out’ the displacement of retinal images on a point-by-point basis [1], [2], current views emphasize the role of cognitive processes such as selective attention and memory, proposing that visual stability depends on updating the representation of few selected elements to match pre- and post-saccadic views [3], [4]. Research has also shown that saccades are accompanied by complex perceptual distortions. Besides an illusory shift of localization for stimuli flashed briefly around saccade onset, there is a compression of the separation among stimuli, both in space [5] and in time [6], as well as an underestimation of the number of elements in the display [7].

It has recently been suggested that magnitude along all three dimensions of space, time and number is encoded within a common abstract metric [8]. Research in numerical cognition provides convergent support for the hypothesis of an abstract magnitude system. Mathematics and the use of numerical systems depend heavily on linguistic abilities and the retrieval of rote memory facts; however, operating with symbolic numerals also relies on manipulating their numerical magnitude, like for non-symbolic representations [9]. For example, choosing the larger of two digits is easier when digits are numerically smaller and farther apart, two general principles governing the discrimination of analogical magnitudes [10], [11]. A fronto-parietal network, involved in the representation of spatial and non-spatial quantities [12], encodes both non-symbolic magnitudes and symbolic numerals [13], [14], and it is active during approximate mental arithmetic [9], [15]. Automatic associations exist between responses to numerical quantity and spatial locations [16], suggesting the idea of a ‘mental number line’, whereby numerical computations exploit the neural machinery of spatial representations.

If physical extent and symbolic quantities impinge on the same abstract representation of magnitude, it is possible that the distortions accompanying saccades also occur when magnitude is just an abstract property of the visual stimuli: symbolic numerical quantity. Here we investigated this possibility by measuring the accuracy of rapidly executed mental additions or subtractions of Arabic numerals. We revealed a transient underestimation of symbolic magnitude, similar to the saccadic underestimation, or compression, of magnitude observed for analogical quantities like space, time and numerosity.

## Materials and Methods

**Figure 1:**
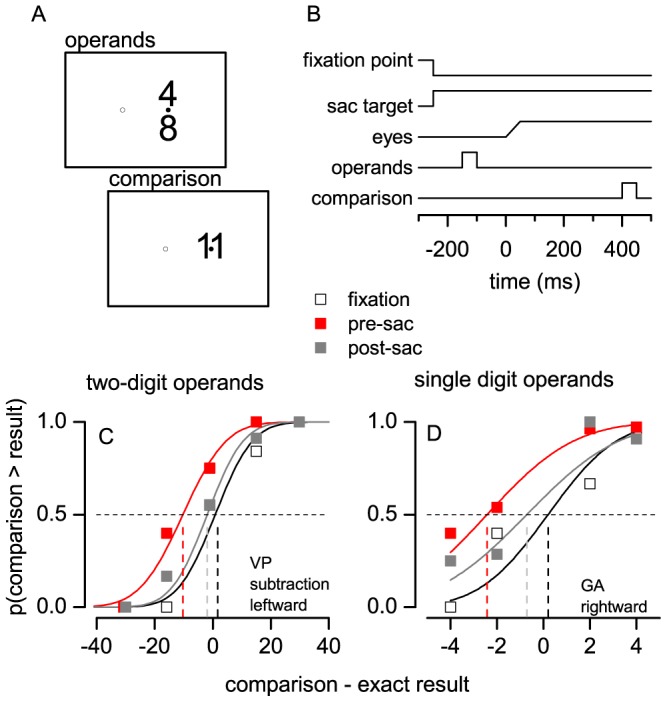
Paradigm and sample results. A–B. Methods. Subjects executed a 20 deg saccade from 10 deg left or right of screen center (hollow and filled circles). Two-digit or single-digit Arabic numerals were briefly presented near the saccade target: the operands and the comparison stimulus, separated by 500 ms. C–D. Example results. The proportion of trials where the comparison stimulus was judged as larger than sum/subtraction of the two-digit (C) or single digit (D) operands is plotted against the difference between the comparison stimulus and the actual result of the operation, yielding psychometric curves for stimuli presented during fixation (black) or between 50 and 250 ms before or after the saccade (red and grey, respectively). Continuous lines are cumulative Gaussian fits; dashed vertical lines indicate the median, or PSE, of each curve.

Two Arabic numerals (‘the operands’) were flashed for 50 ms one above the other, followed after 500 ms by a third flashed numeral (‘the comparison’). Within 1s, subjects reported whether the sum or the subtraction of the operands was smaller or larger than the comparison (using the mouse buttons; an acoustic signal indicated out-of-time responses). Visual stimuli were presented at the target of a saccadic eye movement, which was elicited by a 20 deg displacement of the fixation point (a 0.5 deg black dot; except in control trials where the fixation point remained steady and no saccade was executed). Operands and comparison were large (5×10 deg) and of high contrast (luminance: 0.05 cd/m^2^, against a 30 cd/m^2^ grey background; see Figure 1A).

Experiment 1 tested performance in both addition and subtraction, with both leftward and rightward saccades (in separate sessions). The operands were two-digit numerals, randomly selected so that their sum/subtraction was in the range 30∶60; the comparison was chosen on each trial with the adaptive QUEST procedure [17].

Experiment 2 replicated Experiment 1 with additions of lower and less variable difficulty [based on pilot data and ref. 18]: single digit operands in the range 5∶9, never identical, and with their sum included in the range 11∶15. The comparison stimulus was randomly 4–1 units larger or smaller than the sum of the operands (in 25% catch trials the addends and/or the sum exceeded these limits, so that subjects remained unaware of them). Response time was reduced to 500 ms.

For both experiments, data were analyzed as psychometric curves (Figure 1C–D), plotting the proportion of “sum (subtraction) smaller than comparison” responses, as a function of the difference between the comparison and the exact result of the operation. Data were fit with cumulative Gaussian functions [Psignifit Matlab package, 19] and estimates of the standard errors of the parameters were obtained by Montecarlo simulations (1000 iterations).

Two control experiments employed similar stimuli but no arithmetic task was required. Experiment 3 tested the subjects’ ability to read and recognize the operands and the comparison digits (the comparison stimulus was a one-digit numeral and subjects reported whether it matched one of the “operands”, presented before a rightward saccade or during steady fixation). Experiment 4 tested whether subjects had a bias in reporting the numerical magnitude of individual digits. A single probe digit was presented before a rightward saccade or during steady fixation; subjects reported whether the comparison was numerically larger than the probe. For the control experiments, the response time was reduced further to 400 ms. This ensured that performance remained error-prone even for these relatively easy tasks, avoiding that ceiling effects invalidate the comparison of steady fixation and pre-saccadic conditions. Eight observers (students and laboratory associates, all but two naïve to the aims of the study) volunteered to participate in the experiments: four in Experiments 1, 3 and 4 and five in Experiment 2. All had normal or corrected-to-normal vision and were extensively trained on the mental arithmetic task.

Stimuli were presented on a CRT color monitor (Barco Calibrator, with a screen subtending 60×45 deg at 30 cm distance) driven at a resolution of 464×532 pixels and a refresh rate of 120 Hz by a visual stimulus generator (Cambridge Research Systems VSG2/5) attached to a PC and controlled by Matlab (Mathworks, Natick, MA). Eye movements were recorded by means of an infrared limbus eye tracker (ASL 310). The PC sampled eye position at 1000 Hz, stored the trace in digital form, and estimated the saccadic onset [as described in ref. 7].

Trials were excluded if responses were out of time (14% for Experiment 1, 20% for Experiment 2, 17% for Experiment 3) and if the eye traces revealed unsteady fixation, corrective saccades or saccades that preceded the saccade target presentation (less than 2%), yielding an average 50 trials per subject, condition and stimulus timing.

### Ethics Statement

Before participating in the experiments, subjects gave their written informed consent. Experimental procedures were approved by the local ethics committee (Comitato Etico per la Sperimentazione dei Farmaci, Azienda Ospedaliero-Universitaria Pisana) and are in line with the declaration of Helsinki.

## Results

We asked subjects to compare a rapid estimate of the addition or subtraction of two Arabic numerals (the operands), with a third numeral (Figure 1A–B) presented 0.5 s later. The operands were presented before a saccadic eye movement (between 250 and 50 ms from saccade onset), after its completion, or while subjects maintained steady fixation. We analyzed responses as psychometric curves (Figure 1C–D). The slope of the curves, or JND, estimates the precision of the judgments (i.e. the amount of random errors); their median, or PSE, yields a measure of systematic errors, i.e. tendencies to under- or overestimate the result of the arithmetic operations.

**Figure 2:**
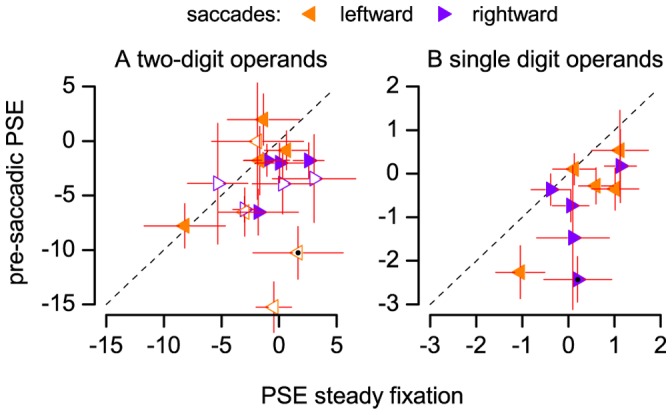
Peri-saccadic systematic errors in a mental arithmetic task. Subjective estimates (PSEs) of additions (filled symbols) and subtractions (hollow symbols) with two-digit (A) or single digit operands (B) presented during fixation (abscissa) or just before a saccade (ordinate). Error bars are standard errors computed by bootstrap. Black dots mark the PSEs of curves in Figure 1C–D.

We found that saccade execution resulted in systematically underestimating additions and subtractions. PSE values for pre-saccadic operands (red curve in Figure 1C) were systematically smaller than PSEs for operands presented during fixation (black) as well as for operands presented after the saccade (grey). Pre-saccadic PSEs for all subjects, saccade direction and operation are plotted against steady fixation PSEs in Figure 2A. The majority of points lay below the equality line; on average, pre-saccadic PSEs were 3.2 units smaller than during fixation, an effect of about 7% of the average correct result of the operation ( = 45). A three-way ANOVA with factors: time from saccade (3 levels: fixation, pre-saccadic, post-saccadic; assumption of sphericity violated and Greenhouse-Geisser correction applied), direction of saccade (leftward, rightward) and operation (sum, subtraction) confirmed that the time of operands presentation relative to the saccade has a significant main effect on PSE values (p<.01). All other main effects and interactions were non significant. The ANOVA did not reveal any systematic effect on JND values (see the slope of the example psychometric curves in Figure 1C–D, approximately constant across conditions), implying that the likelihood of making random errors remained approximately constant across conditions.

Many factors modulate the difficulty of mental arithmetic tasks [18]; for example RTs are smaller for identical numbers (ties) and for small addends (0–1) and longer for carry operations; for two-digit operands these factors can combine to affect task difficulty. We asked whether the pre-saccadic underestimation effect could still be revealed when task difficulty was kept at a relatively constant and low level. We replicated the effect in a second experiment, where subjects performed an addition task with selected single digit operands. The result of the sum was underestimated for operands presented before the saccade (Figure 1D and 2B); the effect was about 1 unit, again about 7% of the average correct result of the operation ( = 13), and it was independent of saccade direction (main effect of the factor: time from saccade, p<0.05; all other main effects and interactions: non significant; no significant effects on JND values).

**Figure 3:**
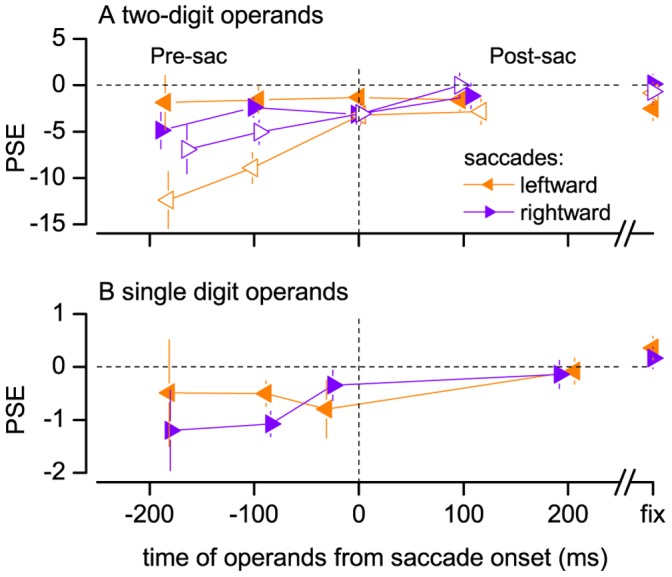
Time-course of systematic errors in a mental arithmetic task. Subjective estimates (PSEs) of mental addition and subtraction for operands presented at variable times from saccade onset (vertical dashed line) and during steady fixation (rightmost values). PSE = 0 means veridical performance (horizontal dashed line). Error bars are standard errors computed by bootstrap. A–B: results for two-digit and single digit operands, respectively.

Figure 3 shows the dynamics of the underestimation effect. PSE values (computed by fitting psychometric curves after pooling data across subjects) are plotted against the time of operands presentation relative to saccade onset. The underestimate is present since the earliest tested time point, i.e. for operands presented soon after the saccade target presentation, and it progressively vanishes as presentation time approaches the saccade onset.

**Figure 4:**
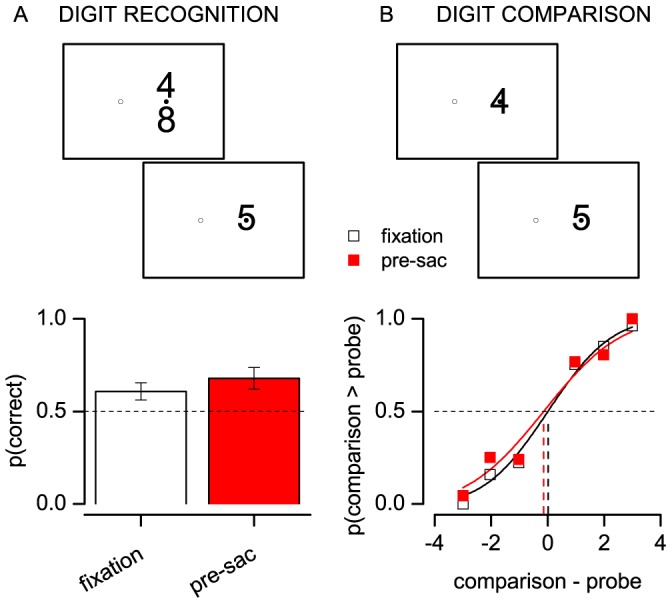
Results of control experiments. Performance on a digit recognition task (A), averaged across subjects (proportion of trials where subjects correctly reported if the comparison stimulus matched one of the operands, presented during fixation or before a rightward saccade; error bars are standard errors of the mean). Results of the digit comparison task (B), pooled across subjects (proportion of trials where the comparison was judged as larger than a single-digit probe, plotted as function of the numerical distance between the two numerals, and fit with a cumulative Gaussian).

In order to demonstrate that saccade execution did not generically impair sensory processing of the numerals, we conducted a control experiment where subjects performed no mental arithmetic task, but simply reported whether the comparison stimulus (a single digit numeral) was identical to one of the “operands”. Percentage correct was not significantly different before a saccade and in steady fixation conditions (Figure 4A, two-tailed paired samples t-test, p>0.1), indicating that the pre-saccadic stimuli were normally recognized. In a final experiment, we showed that the pre-saccadic underestimate observed in the mental arithmetic task could not be explained by an underestimate of the individual operands. Subjects reported which of two sequentially presented digits was larger (the time-course of presentations was the same as in the main experiments). Figure 4B shows the psychometric functions resulting from plotting the proportion of “comparison larger than probe” responses as a function of the numerical distance between the stimuli (data pooled across the four tested subjects). Although there was a trend for pre-saccadic PSEs to be smaller for pre-saccadic probes than during steady fixation (the average PSE was −0.45±0.5 for pre-saccadic probes and −0.04±0.13 in steady fixation), the difference was not statistically significant (two-tailed paired sample t-test on the PSEs of psychometric curves for the individual subjects: p>0.1). Note that the allowed response time was shorter in the control experiments (400 ms) than in the mental arithmetic tasks (1000 and 500 ms for the two-digit and single-digit operations), preventing the possibility to compare the proportion of encoding errors across experiments.

## Discussion

We report the occurrence of systematic errors in performing rapid mental additions and subtractions before the execution of a saccade: an underestimate of numerical magnitude. This effect parallels our previous findings with analogical magnitude and non-symbolic numerals [20].

We find that, just before but not just after saccades, subjects underestimate the result of arithmetic operations. Saccades did not significantly affect performance in a single-digit inequality judgment, suggesting that, for a reliable pre-saccadic number underestimation to be revealed, subjects needed to be involved in a challenging task that forced them to manipulate numerical magnitudes. For the mental arithmetic tasks, the underestimation effect was about 7% for both two-digit and single digit operands. Previous observations with non-symbolic numerals revealed a larger underestimate, about 35% of the cardinality of the set [7]. This fits well with the idea that, while symbolic and non-symbolic numerals are processed by partially shared mechanisms, symbolic numerals are encoded with greater precision [13], [14], [21]. The effect we report here occurs within a large pre-saccadic temporal window (Figure 3), whereas the underestimate of non-symbolic numerosity [7] unfolds with a faster dynamics, peaking at saccadic onset and starting some 50 ms before. This discrepancy may depend on the long and variable processing times for symbolic numerals [22], which is consistent with an effect of saccade execution on stimuli presented within a wide temporal window, starting long before the saccade.

Previous work has demonstrated effects of gaze position [23] and gaze shifts [24], [25] on responses to numerals. Our study is distinct from these, and our results are novel, in two important ways.

First, our results cannot be accounted for by a generic deployment of processing resources – a feasible explanation for previously reported effects of saccades on RTs in a digit comparison task [24]. Two observations support this claim: that we did not observe an increase in overall error rate, and that the recognition of the numerals was not affected by the saccade. Note that our paradigm was designed to minimize the deployment of attentional resources to the execution of the saccade task, by positioning stimuli in the region of the saccade target, where attention is allocated before a saccade [26]. While a dual-task cost cannot explain the observed systematic underestimation we observe, without an accompanying change of JNDs in the mental arithmetic task and without an increase of the overall error rate in the control tasks, further research is needed to test whether the pre-saccadic underestimation effect observed here can also be induced by manipulating covert attention, which can induce distortions similar to those observed around the time of saccades [27].

Second, given that the underestimation effect was observed irrespectively of the direction of saccades and the type of arithmetic operation, it cannot be explained by an association between responses to small/large numbers and the left/right space [the ‘SNARC’ effect, 16] or a tendency to associate large/small numbers to the operations of addition/subtraction (the Operational Momentum effect [28]).

Although independent from the SNARC effect, our results are consistent with the hypothesis of a link between the processing of abstract numerical quantities and the representation of magnitude along multiple dimensions: not only number, but also space and time [8], [12], [20]. Imaging and neuropsychological evidence points to the intra-parietal cortex as a pivotal area for an abstract representation of magnitude [8], [29]. Interestingly, the same areas are involved in the preparation of eye movements [30] and in the maintenance of perceptual stability [31], and this may explain the concurrent distortions of magnitude judgments observed in the proximity of saccades [20].While the existence and format of such abstract magnitude representation is still a matter of speculation, current computational approaches of numerical cognition indicate that a link between symbolic and non-symbolic magnitude is critical to explain arithmetic performance in normal subjects and in clinical populations [32]. In a recent proposal [33], the presentation of a symbolic arithmetic problem automatically activates analogical numerosity representations, by progressively recruiting units that represent increasing magnitudes. It is interesting to note that, reducing processing time or resources in this network should result in a systematic underestimation of magnitude for both symbolic and non-symbolic quantities.

In conclusion, the present experiments demonstrate that mental arithmetic is impaired before the execution of saccadic eye movements. These results reinforce the hypothesis of shared mechanisms supporting the representation of symbolic and non-symbolic quantities, and they establish a surprising link between the preparation of actions and the processing of abstract quantities.
